# Functionalized TMC and ε-CL elastomers with shape memory and self-healing properties

**DOI:** 10.3389/fbioe.2023.1298723

**Published:** 2023-11-16

**Authors:** Siwen Chen, Miaomiao Xiao, Zhipeng Hou, Zhongcun Li, Jianshe Hu, Jing Guo, Jing Chen, Liqun Yang, Quan Na

**Affiliations:** ^1^ Research Center for Biomedical Materials, Engineering Research Center of Ministry of Education for Minimally Invasive Gastrointestinal Endoscopic Techniques, Shengjing Hospital of China Medical University, Shenyang, China; ^2^ Center for Molecular Science and Engineering, College of Science, Northeastern University, Shenyang, China; ^3^ College of Kinesiology, Shenyang Sport University, Shenyang, China; ^4^ Liaoning Research Institute for Eugenic Birth and Fertility, China Medical University, Shenyang, China; ^5^ Department of Obstetrics and Gynecology, Shengjing Hospital of China Medical University, Shenyang, China

**Keywords:** elastomer, polycarbonate, polyester, self-healing, shape recovery

## Abstract

**Introduction:** Smart elastomers, which possess self-healing and shape memory capabilities, have immense potential in the field of biomedical applications. Polycarbonates and polyesters have gained widespread interest due to their remarkable biocompatibility over the last century. Nevertheless, the lack of functional versatility in conventional polyesters and polycarbonates means that they fall short of meeting the ever-evolving demands of the future.

**Methods:** This paper introduced a new smart elastomer, named mPEG_43_-*b*-(PMBC-co-PCL)_n_, developed from polyester and polycarbonate blends, that possessed shape memory and self-heal capabilities via a physical crosslinking system.

**Results:** The material demonstrated a significant tensile strength of 0.38 MPa and a tensile ratio of 1155.6%, highlighting its favorable mechanical properties. In addition, a conspicuous shape retrieval rate of 93% was showcased within 32.5 seconds at 37°C. Remarkably, the affected area could be repaired proficiently with no irritation experienced during 6h at room temperature, which was indicative of an admirable repair percentage of 87.6%. Furthermore, these features could be precisely modified by altering the proportion of MBC and ε-CL to suit individual constraints.

**Discussion:** This innovative elastomer with exceptional shape memory and self-heal capabilities provides a solid basis and promising potential for the development of self-contracting intelligent surgical sutures in the biomedical field.

## 1 Introduction

In practical applications, elastomers are frequently exposed to external factors such as environmental conditions and external stresses. These exposures can result in external damage, fractures, or internal cracking, ultimately leading to material failure (Utrera-Barrios et al., 2022). Most elastomers lack self-healing properties, and once the material is damaged, it cannot be reshaped, often resulting in significant waste (Wemyss et al., 2020). Self-healing elastomers have the capability to restore their structural integrity under specific conditions following material damage (Wang and Urban, 2020). Self-healing elastomers are typically divided into exogenous and intrinsic types (White et al.; Guo et al., 2020). The self-healing capacity of exogenous self-healing elastomers is restricted by the pre-embedded repair reagents within the material. Once these repair reagents are exhausted, the material loses its self-healing ability (Kanu et al., 2019). Intrinsic self-healing elastomers derive their self-healing ability from the breakage and reorganization of reversible chemical bonds in dynamic cross-linked networks, such as hydrogen bonds, ionic interactions, metal-ligand coordination, disulfide exchange, and Diels–Alder reactions (Jian et al., 2018; Song et al., 2018; Fan et al., 2019; Li et al., 2020; Wang et al., 2020; Yang et al., 2020). Compared to exogenous self-healing elastomers, intrinsic self-healing elastomers possess the advantage of an unlimited number of repair cycles, as the repair reagent does not impose limitations. In addition to their self-healing capabilities, these elastomers generally exhibit a higher Young’s modulus and stress, greater elongation at break, and enhanced heat resistance compared to similar non-self-repairing elastomers (Xie et al., 2021).

Shape Memory Elastomers (SMEs) belong to a category of smart materials known for their capability to revert from a temporarily programmed shape to their original permanent shape in response to applied forces and external stimuli, such as temperature, pH, light, water or solvents, as well as electric and magnetic fields, among others (Lapcık et al., 1998; Liu et al., 2009; Han et al., 2012; Qi et al., 2014; Bai and Shi, 2017; Zhao et al., 2017; Kong et al., 2019; Davidson et al., 2020; Ze et al., 2020; Cui et al., 2021). Briefly, SMEs are exposed to an external stimulus, typically by heating them above the glass transition temperature while applying an external force. This is followed by cooling below the glass transition temperature for temporary programming, and finally, returning to their original permanent shape upon heating to an appropriate temperature (Chen et al., 2020). Permanent shapes are typically established through covalent bonding, whereas temporarily programmed shapes are generally achieved through weak interactions such as hydrogen bonding, hydrophobic interactions, π-π superposition, ionic bonding, host-guest interactions, etc (Jiang et al., 2017; Wu et al., 2020; Chen Z. et al., 2021; Gallos et al., 2021). Among them, thermally induced shape memory elastomers have garnered significant attention from researchers in recent years, owing to their high deformation rates and ease of production techniques (Chen et al., 2020; Zhao et al., 2020; Yin et al., 2022).

Surgical sutures are one of the most frequently utilized medical devices in daily practice, aimed at facilitating wound healing and minimizing scarring through the secure joining of body tissues. The ideal surgical suture should have good biocompatibility, appropriate mechanical properties, low inflammatory response, and ease of knotting (Shao et al., 2016; Alshomer et al., 2017). The primary challenge encountered with surgical sutures lies in their limited maneuverability during specific minimally invasive procedures for wound closure, posing difficulties in achieving secure suturing and knotting. Moreover, inadequate suture strength can result in visible scar tissue at the wound site, potentially causing infections and hernias. Conversely, excessive suture strength may escalate patient discomfort and induce tissue necrosis at the wound site (Sheng et al., 2017; Tsukamoto et al., 2018; Chen et al., 2022). The utilization of SMEs as a promising choice for surgical sutures represents an appealing strategy. SMEs equipped with temperature-sensitive triggers can undergo solidification at lower temperatures, effectively storing internal stresses, and subsequently tightening the suture by releasing these stresses at body temperature (Duarah et al., 2018). Therefore, surgical sutures crafted using temperature-sensitive SMEs hold great potential for enhancing surgical maneuvers.

Trimethylene carbonate (TMC) andε-CL are common polymer monomers within the biomedical field. The copolymerization of these monomers exhibits excellent biocompatibility and has been the subject of extensive research in recent years (Mathot et al., 2007; Danhier et al., 2009; Yang et al., 2014a; Yang et al., 2014b; Pires et al., 2016). The conventional P (TMC-co-CL)_n_ lacks self-healing and shape memory capabilities, thereby limiting its potential applications. In materials science, functionalized modifications to monomers are a prevalent approach to bestow polymers with superior properties (Xu et al., 2014; Ansari et al., 2021). The copolymer’s potential for biomedical applications could be significantly enhanced by incorporating ambient temperature self-healing properties and shape memory functionality into P (TMC-co-CL)_n_ using this method.

In this investigation, we presented a novel shape memory elastomer with self-healing properties, denoted as mPEG_43_-*b*-P (MBC-co-CL)_n_, obtained by copolymerization of the TMC-functionalised monomer MBC and ε-CL. The elastomer was synthesized using mPEG_43_ as the macromolecular initiator, MBC and CL as the functional monomers, and Sn(Oct)_2_ as the reaction catalyst for the ring-opening polymerization reaction. Due to the introduction of benzyl carbonyl groups in MBC, mPEG_43_-*b*-P (MBC-co-CL)_n_ became insoluble in dichloromethane. Infrared analysis confirmed the formation of an internally structured physical crosslinked network within the elastomer, created by hydroxyl and carbonyl groups interacting to form hydrogen bonds. The elastomer mPEG_43_-*b*-P (MBC-co-CL)_n_ exhibited excellent mechanical properties and stretchability. It automatically healed wounds at room temperature without external stimulation and simultaneously demonstrated impressive shape memory at human body temperature. These functions could be regulated by adjusting the ratio of MBC to ε-CL, wherein self-healing capability and shape recovery rate increased with higher MBC content. Compared to P (TMC-co-CL)_n_, the elastomer mPEG_43_-*b*-P (MBC-co-CL)_n_ demonstrated enhanced self-healing and shape memory functions, thus broadening the potential applications of P (TMC-co-CL)_n_ in the biomedical field. Compared to traditional surgical sutures, the mPEG_43_-*b*-P (MBC-co-CL)_n_ elastomer exhibits excellent self-healing and shape memory properties at room temperature, with the potential to create knotless self-tightening surgical sutures. This advancement opens up prospects for its potential use in self-retracting surgical suture applications.

## 2 Experimental section

### 2.1 Materials

2,2-Bis(hydroxymethyl) propionic acid (99%) was bought from Tianjin Bodi Chemical Co., Ltd. (Tianjin, China). Toluene (99%), Calcium hydride (CaH_2_), and Benzyl chloride (99%) were purchased from Sinopharm Chemical Reagent Co. Ltd. (Shanghai, China). Triethylamine (99%) and tetrahydrofuran were purchased from Tianjin Damao Chemical Reagent Factory. (Tianjin, China). Methoxy polyethylene glycol (mPEG_43_, 1.9 kDa) was purchased from InnoChem Technology Co. Ltd. (Beijing, China). ε-CL and Stannous octoate [Sn(Oct)_2_] (99%) were supplied by Sigma-Aldrich. Ethyl chloroformate (99%) was purchased from Xinyi Huili Fine Chemical Co., Ltd. (Xinyi, China). The MBC is synthesized according to a previously published procedure (Liu et al., 2003). ε-CL was stirred with CaH_2_ for 2 days to dry and then distilled under reduced pressure. 5-methyl-5-benzyloxycarbonyl-1,3-dioxan-2-one (MBC) was synthesized based on previous work (Chen et al., 2020). Toluene was de-watered by sodium filament azeotropic reflux before use. Standard methods purified all other solvents in this study.

### 2.2 Synthesis of the elastomer mPEG_43_-*b*-P (MBC-co-CL)_n_

Utilizing mPEG_43_ as the initiator for ring-opening polymerization and Sn(Oct)_2_ as the catalyst, the ratios of monomer MBC and CL were determined as 1:1, 3:1, and 5:1. The ratio of monomer to the initiator (M:I) was set to 2,000:1 and the ratio of monomer to catalyst (M:C) was given at 1,000:1. These combinations resulted in three distinct ratios of elastomers named M_1_, M_2_, and M_3_, with specific details outlined in Table 1. In summary, the polymerization cross-linking process began by placing the PTFE tube mold (length 30 mm, inner diameter 2 mm) inside an ampoule. Subsequently, a toluene solution (0.2 mol/L) containing the monomer, mPEG_43_ and Sn(Oct)_2_ was added to the ampoule. The system was vacuumed and purged with nitrogen three times before the ampoule was sealed to maintain a vacuum-tight system (<15 Pa). Afterward, the ampoule was positioned in an oil bath container at 130°C for 24 h. The elastomers underwent a 72 h immersion in dichloromethane (DCM) to exclude polymer chain segments not involved in cross-linking, including any unreacted monomers. Afterwards, they were vacuum-dried for 24 h until a stable weight was attained.

**TABLE 1:** Reaction conditions for the elastomer mPEG_43_-*b*-P (MBC-co-CL).

Samples	MBC: ε-CL	M: I	M: C	T (^o^C)	t(h)
M_1_	1:1	2000:1	1,000:1	130	24
M_2_	3:1	2000:1	1,000:1	130	24
M_3_	5:1	2000:1	1,000:1	130	24

### 2.3 Characterization

Fourier transform infrared spectra (FT-IR) was acquired using a PerkinElmer Spectrum One (B) Spectrometer (PerkinElmer, Foster City, CA, USA) over the range of 4,000–500 cm^-1^ at room temperature.

Thermogravimetric analysis (TGA) was conducted using a Netzsch 209C TGA instrument (Netzsch, Hanau, Germany) over the temperature range of 40°C–800°C with a ramp rate of 20°C/min under nitrogen purging. Differential scanning calorimetry (DSC) was performed utilizing a NETZSCH DSC-204 thermal analyzer (Netzsch, Hanau, Germany). Data was recorded within the temperature range of −50°C–200°C at a rate of 10°C/min under a flowing nitrogen atmosphere.

The swelling experiments were carried out in water and dichloromethane. The weighed samples were immersed in the solvent and changed the solvent every 24 h for a total of 72 h. After removing and drying the surface solvent, the samples were weighed (W_1_) and vacuum-dried to constant weight (W_2_). The swelling rate (SR) of the elastomer was calculated by Eq. 1, and the gel fraction (GF) was calculated by Eq. 2.

$$SR=\frac{W_{1}-W_{0}}{W_{0}}\times 100\%$$ (1)

$$GF=\frac{W_{2}}{W_{0}}\times 100\%$$ (2)

W_0_: Initial weight of the elastomer.

A dynamic mechanical analyzer (242E Artemis DMA, Netzsch, Germany) was employed to assess the viscoelastic properties of the polymer. During all tests, measurements were conducted in the tensile mode using cylindrical samples measuring 2 mm in diameter and 20 mm in length. The storage modulus (E′) and loss modulus (E″) of the polymers were measured at 25°C over a frequency range of 0.1–100 Hz for frequency testing and at a constant frequency of 1 Hz for various temperatures ranging from 25°C to 70°C. Three replicates were carried out for each sample scale. Tensile mechanical properties of the elastomer were evaluated using the MTS MCT-6103 (Meister, China) universal mechanical testing machine. Elastomeric rods, 20 mm in length and 2 mm in diameter were tested at room temperature with a clamping distance of 10 mm and a stretching rate of 10 mm/min.

The investigation into the self-healing properties of elastomers involved cutting a rod-shaped elastomer in half and rejoining the two cut surfaces, allowing them to contact and self-heal at 25°C for a specified duration. The repaired elastomer was then examined under an optical microscope (Smartzoom 5) to document alterations in the self-healing scratch trajectory. Subsequently, mechanical properties were evaluated using a universal testing machine.

The assessment of the elastomer’s shape memory capability proceeded as follows: an external force was applied to the I-shaped rod elastomer at room temperature, causing it to adopt a U-shape (temporary form). Subsequently, the elastomer was solidified in a −20°C environment. Finally, the elastomer was transferred to a 37°C environment, reverting it to its original I-shape (permanent form). The entire process was meticulously recorded using a video camera. Shape recovery rate (R_f_) and shape fixation rate (R_r_) were calculated by Eqs 3, 4. The shape memory measurement process is shown in Figure 6A.

$$R_{f}=\frac{{180-\alpha }_{0}}{180}\times 100\%$$ (3)

$$R_{r}=\frac{{180-\alpha }_{0}-\alpha_{t}}{180-\alpha_{0}}\times 100\%$$ (4)

α_0_: The angle of the elastomer at initial fixation. α_t_: The angle of the elastomer at time t.

## 3 Result and discussion

### 3.1 Structural characterization of elastomers

**FIGURE 1:**
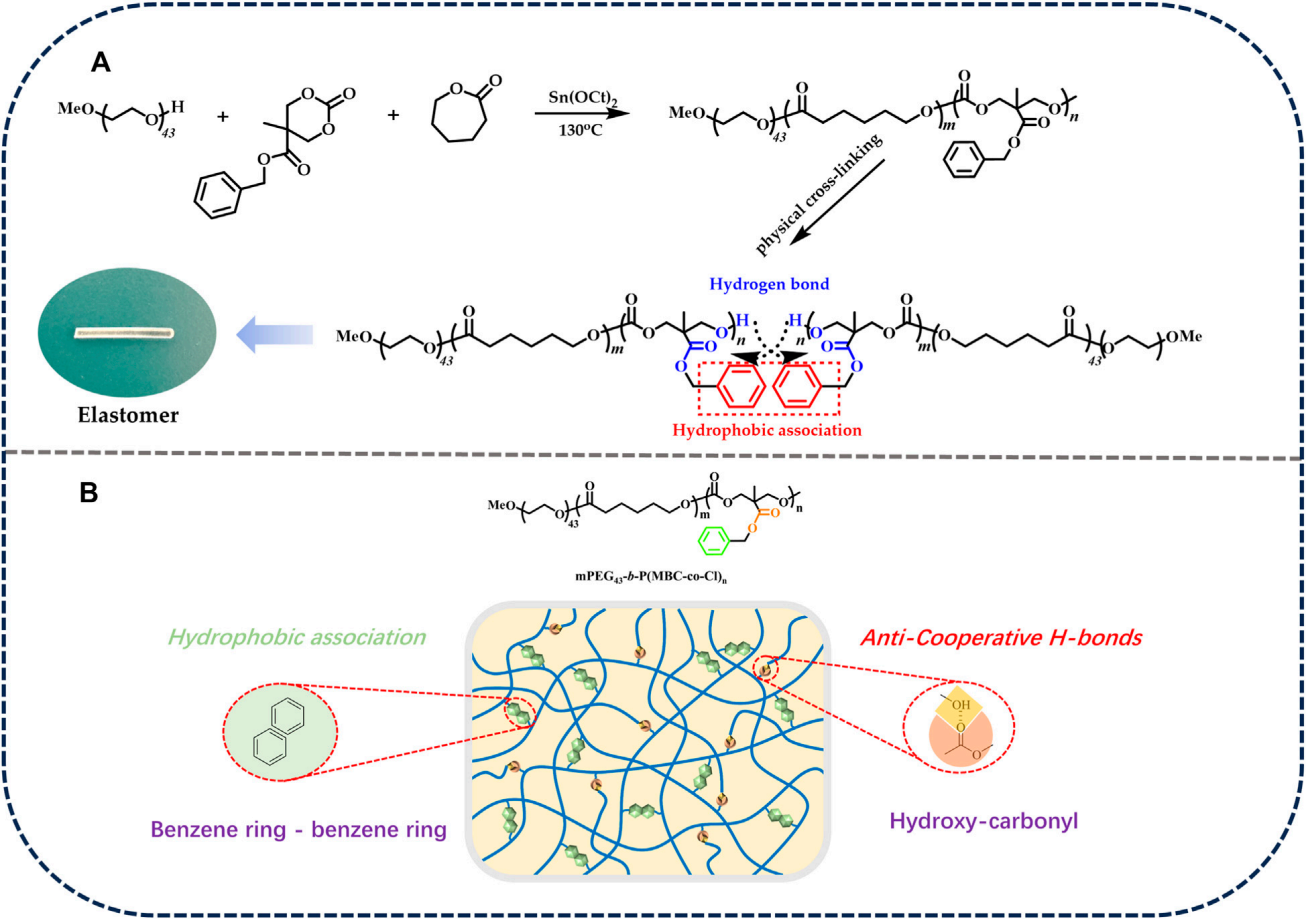
**(A)** Ring-opening polymerization of elastomers. **(B)** The formation of physically crosslinked elastomers.

The polymerization conditions of the elastomers are shown in Table 1. The process of the ring-opening polymerization reaction and the formation of physically crosslinked elastomers are shown in Figure 1. The structures of the elastomers were analyzed by FT-IR.

**FIGURE 2:**
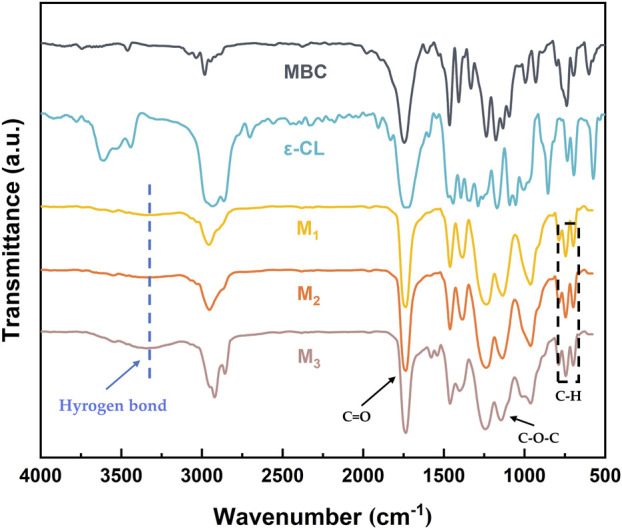
The FT-IR spectrum of monomers and elastomers (M_1_, M_2_, and M_3_).

The FT-IR of the elastomer is shown in Figure 2. The mPEG-*b*-(PMBC-co-PCL)_n_ copolymer was synthesized by initiating the ring-opening polymerization of MBC and ε-CL by using mPEG_43_ as initiator and Sn(Oct)_2_ as catalyst. The C-H stretching vibrations of 3,100–3,000 cm^−1^ and the out-of-plane bending vibrations of C-H of 698 and 744 cm^−1^ proved the existence of the benzene ring. The stretching vibrations at 1,737 and 1,134 cm^−1^ were the ester carbonyl and aliphatic ether bonds of the copolymer, which demonstrated the successful participation of MBC and ε-CL in the ring-opening polymerization reaction. The broad peaks appearing at 3,500–3,150 cm^−1^ in the FT-IR spectrum of M_1_, M_2_, and M_3_ were due to the hydrogen bonding reaction between the hydroxyl group at the end of the polymer and the ester carbonyl group of the PMBC side chain (Chen C. et al., 2021). The gradual enhancement of the hydrogen bonding peaks of M_1_, M_2_, and M_3_ was attributed to the increase in the MBC content which increased the probability of hydrogen bond formation.

### 3.2 Thermodynamic properties of elastomers

**FIGURE 3:**
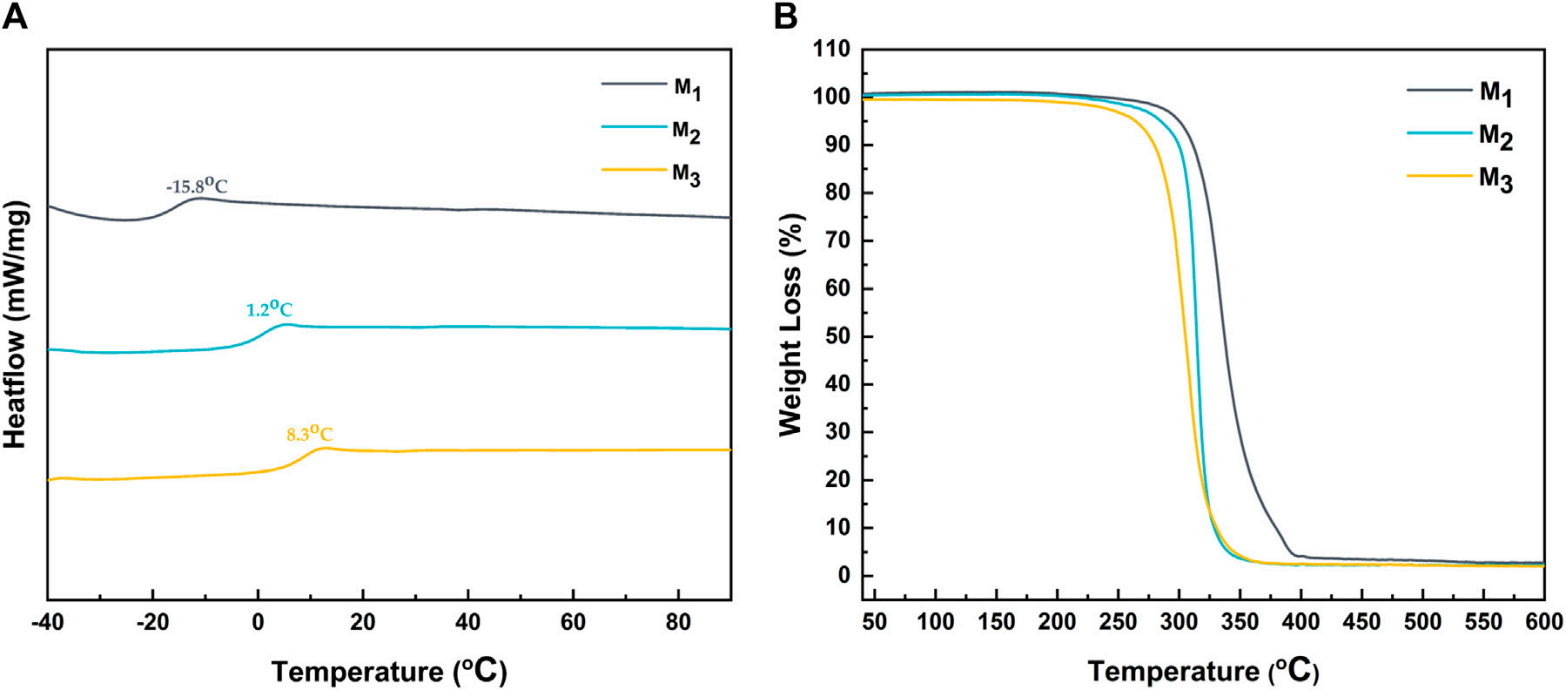
The **(A)** DSC and **(B)** TGA of elastomers.

The glass transition temperature analysis of the elastomer was performed using DSC. As illustrated in Figure 3A, there was a notable increase in the glass transition temperature of the elastomer with the augmentation of MBC content, ranging from −15.8°C for M_1_ to 8.3°C for M_3_. This phenomenon could be attributed to the increase in the number of benzene rings and side chain ester carbonyls in MBC, elevating the likelihood of π-π conjugation between benzene rings and the formation of both intramolecular and intermolecular hydrogen bonding within the system. Additionally, a decreased ε-CL ratio resulted in fewer flexible chain segments in the molecule, consequently enhancing the overall rigidity of the elastomer. Furthermore, TGA characterization was performed to analyze the thermal behavior of the elastomers.

Figure 3B demonstrated the weight loss of the elastomer at various temperatures, and the temperature at which the elastomer experiences a 5% weight loss is defined as its decomposition temperature. Interestingly, the decomposition temperature decreased with an increase in MBC content. This was due to the fact that the decomposition temperature of PMBC is lower than that of PCL. Specifically, the decomposition temperature for M_1_, M_2_, and M_3_ were determined to be 300.1°C, 285°C, and 264.3°C, respectively.

### 3.3 Swelling properties of elastomers

**FIGURE 4:**
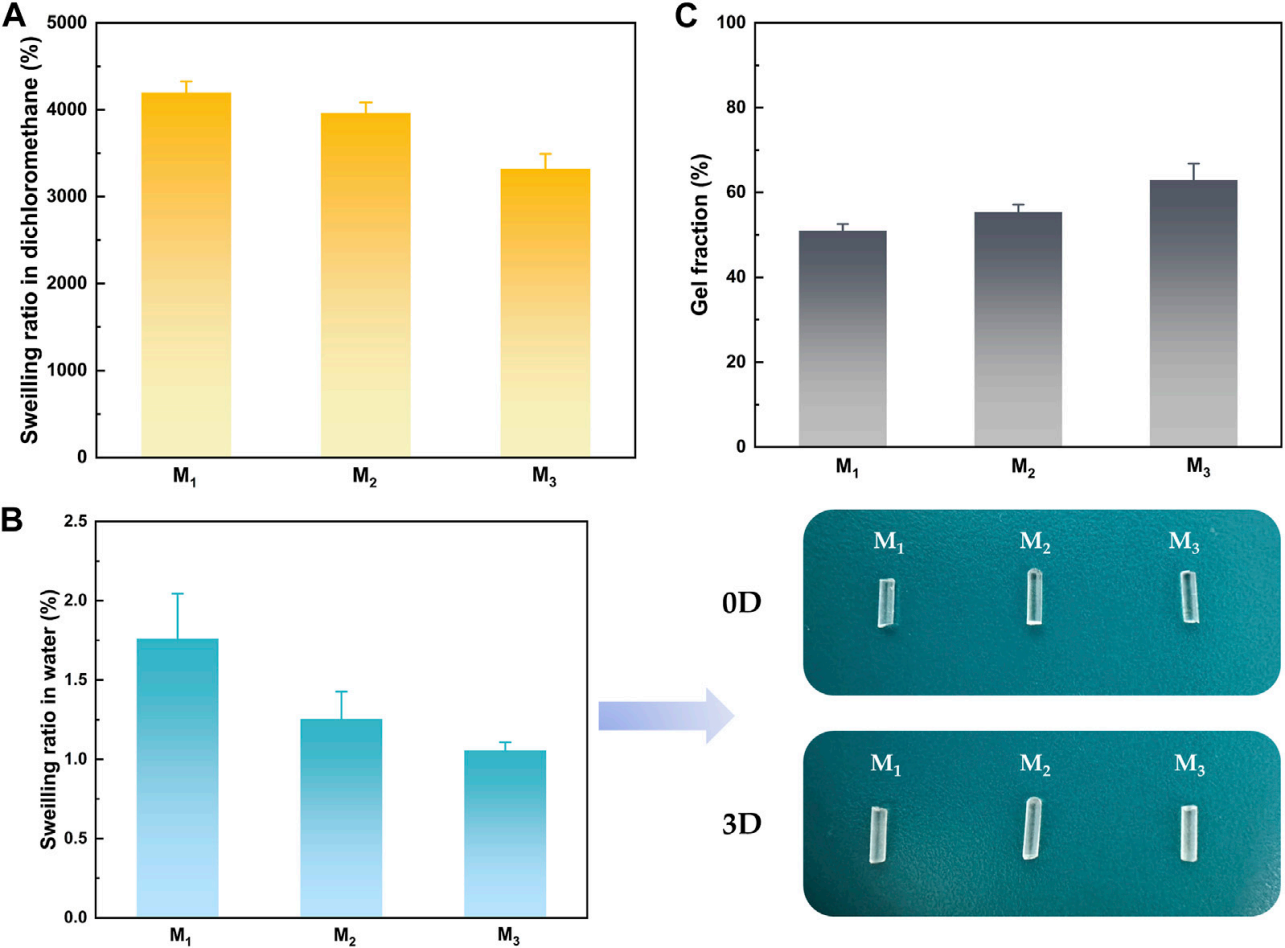
Solubilization properties of elastomers in **(A)** dichloromethane **(B)** water, and **(C)** gel fraction.

The impact of MBC content on the crosslinking network was revealed through the analysis of the swelling behavior of elastomers in water and organic solvents with three distinct MBC ratios. Based on the data presented in Figures 4A, B, it was observed that as the MBC content increased, the swelling ratio of the elastomer in both dichloromethane and water decreased. As the MBC content in the crosslinked network rose, there was an increase in π-π conjugation between the benzene rings and an elevated quantity of hydrogen bonds between the hydroxyl groups at the polymer segment termini and the ester carbonyls of the side chains, leading to an increase in the degree of cross-linking. Furthermore, the benzene ring, classified as a lipophilic group, played a role in enhancing the hydrophobicity of the elastomer due to an increased presence of benzene rings within the polymer chain segments. This heightened hydrophobicity was evidenced by the gel fraction of the elastomers depicted in Figure 4C, showcasing an augmentation with the rise in MBC content. The escalation of MBC content facilitated an increase in the number of hydrogen bonds within the crosslinked network. Consequently, intermolecular and intramolecular entanglements were more readily formed, contributing to the heightened gel fraction of the elastomers. Elastomers are virtually insoluble in water, so there was no change in properties in the body due to fluid exchange. Therefore, the elastomers held promising potential for their application in surgical suture direction.

### 3.4 Dynamic thermo-mechanical properties

**FIGURE 5:**
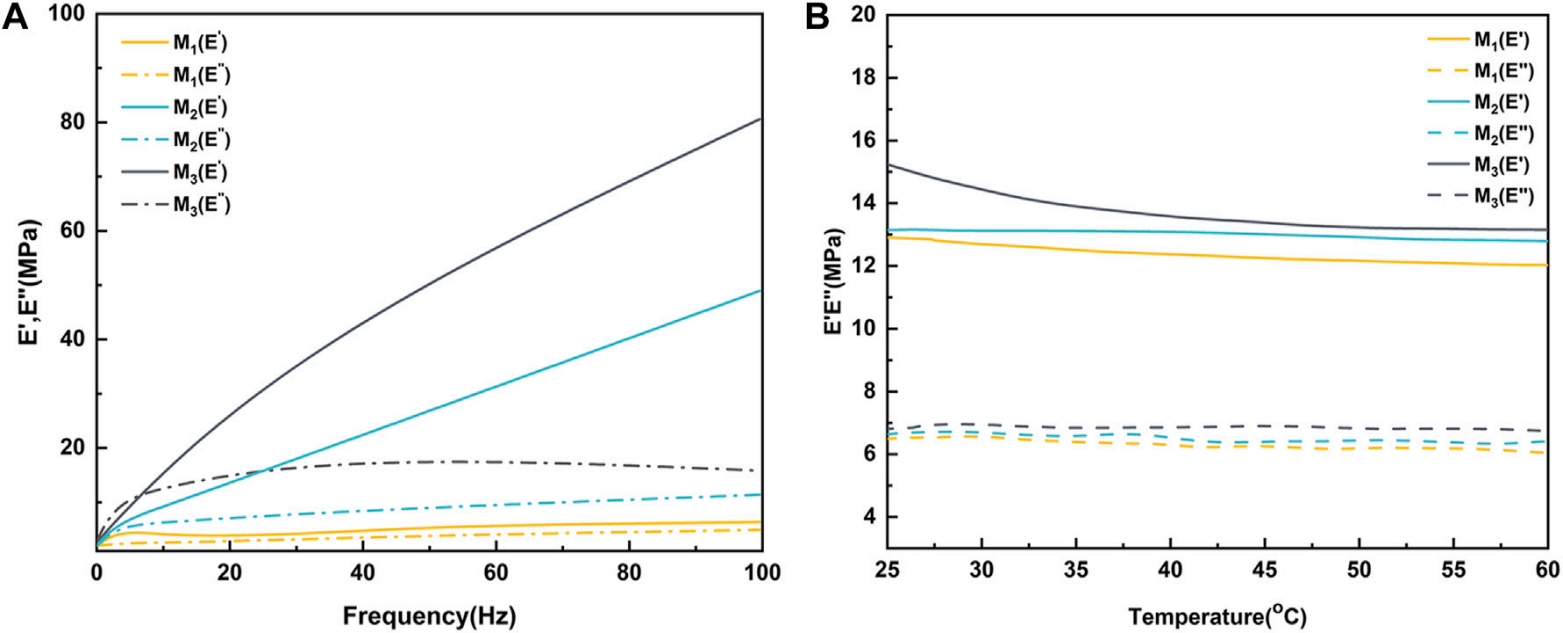
Dynamic thermo-mechanical properties of elastomers with **(A)** variable frequency at room temperature and **(B)** variable temperature at 1 Hz.

The dynamic thermo-mechanical properties of the elastomers are illustrated in Figure 5. In the frequency sweep test at room temperature (Figure 5A), M_1_ displayed the highest energy storage modulus (E′) at 6.3 MPa and a loss modulus (E″) of 4.9 MPa at 100 Hz. Across all samples, there was a noticeable trend of increasing E′ and E″ with higher frequencies. Additionally, both E′ and E″ of the elastomers exhibited a significant upward trend with increasing MBC content. Specifically, for M_3_, the values were measured at 80.6 MPa (E′) and 15.82 MPa (E″), respectively. This phenomenon could be attributed to the increase in MBC content, resulting in a higher number of side-chain hydroxyl groups and benzene rings in the molecular chain segments. This increase enhanced the likelihood of hydrogen bonding, π-π stacking formation, and an overall rise in crosslink density. Beyond the frequency sweep test, a fixed 1 Hz variable temperature test was conducted on the elastomers (Figure 5B). It was observed that regardless of the monomer ratio, the E′ and E″ of the elastomers decreased with increasing temperature. This could be explained by heightened molecular thermal motion at high temperatures, causing an increase in distance between the molecules of the chain segments and reducing the effects of hydrogen bonding and π-π stacking. This outcome suggested that elastomers tend to exhibit higher viscosity at elevated temperatures, potentially enhancing their self-healing properties.

### 3.5 Shape memory behaviors and self-healing properties

**FIGURE 6:**
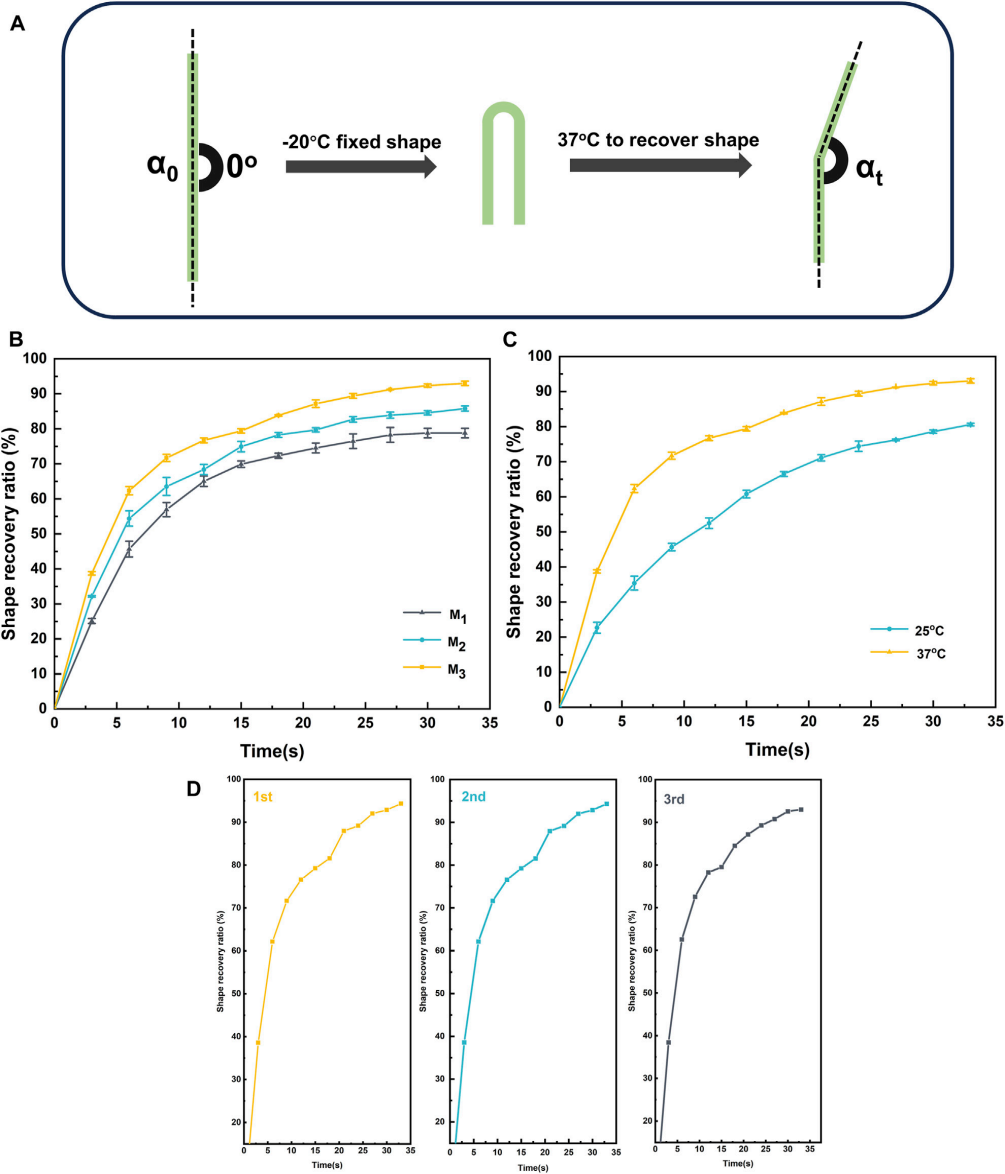
Shape memory **(A)** processes of elastomers with **(B)** different monomer ratios , **(C)** different temperatures of M_3_ and **(D)** shape memory repeatability of M_3_.

Elastomers could be endowed with shape memory properties utilizing a dynamic crosslinked network structure and a glass transition temperature (T_g_). Initially, the elastomer was shaped into a temporary form at room temperature and subsequently solidified in an environment below T_g_. Later, when exposed to a 37°C environment, the molecular motion within the elastomer’s crosslinked network was reactivated, and the rigidity was induced to revert to the initial shape. This transformation was facilitated by the increased rigidity effect resulting from hydrogen bonding and π-π stacking. Figure 6A illustrated the shape memory process of the elastomer. Figure 6B illustrated the deformation recovery at various recovery times for the elastomer samples denoted as M_1_, M_2_, and M_3_. The deformation recovery for M_1_, M_2_, and M_3_ was observed to be completed within 32.5 s at 37°C, displaying recovery rates (R_r_) of 87.2% and remarkable restoration rates (R_f_) of 93% (Table 2). As the MBC content increased in the elastomer, there was a notable decrease in Rf, although Rr exhibited a significant increase. This behavior stemmed from the interplay of MBC content with the elastomer’s rigidity and interaction forces within the rigid chain segments. Specifically, at lower MBC content, the interaction force among the rigid chain segments in the elastomer was relatively weak, resulting in a gel with a feeble restoring force. Conversely, higher MBC content intensified hydrogen bonding and π-π stacking in the crosslinked network, enhancing the elastomer’s rigidity, inducing substantial phase separation, and making shape fixation less facile, yet augmenting repulsive forces. Furthermore, we conducted a study on the shape memory functionality of the elastomer at different temperatures. As shown in Figure 6C, at 37°C, the elastomer exhibited a significantly higher R_f_ (93%) compared to the R_f_ (80.6%) observed at 25°C. This is attributed to the elevated temperature, which accelerates the motion of polymer chain segments within the elastomer, resulting in a faster shape recovery. In addition to the analysis of Rf and Rr, evaluating the repeatability and fatigue resistance of elastomer shape memory was paramount for assessing its potential in practical applications. Using M_3_ as a representative, as depicted in Figure 6D, we examined the shape memory capability of the elastomer over three consecutive cycles. Remarkably, the same sample exhibited no signs of fatigue and maintained a consistently stable shape memory capacity throughout the three consecutive cycles. This observation underscored the elastomer’s robust fatigue resistance and its capability to sustain a reliable shape memory performance over subsequent practical applications.

**TABLE 2:** Shape memory properties of M_1_, M_2_, and M_3_.

Samples	R_r_ (%)	R_f_ (%)
M_1_	95.2	78.8
M_2_	92.1	85.8
M_3_	87.2	93

**FIGURE 7:**
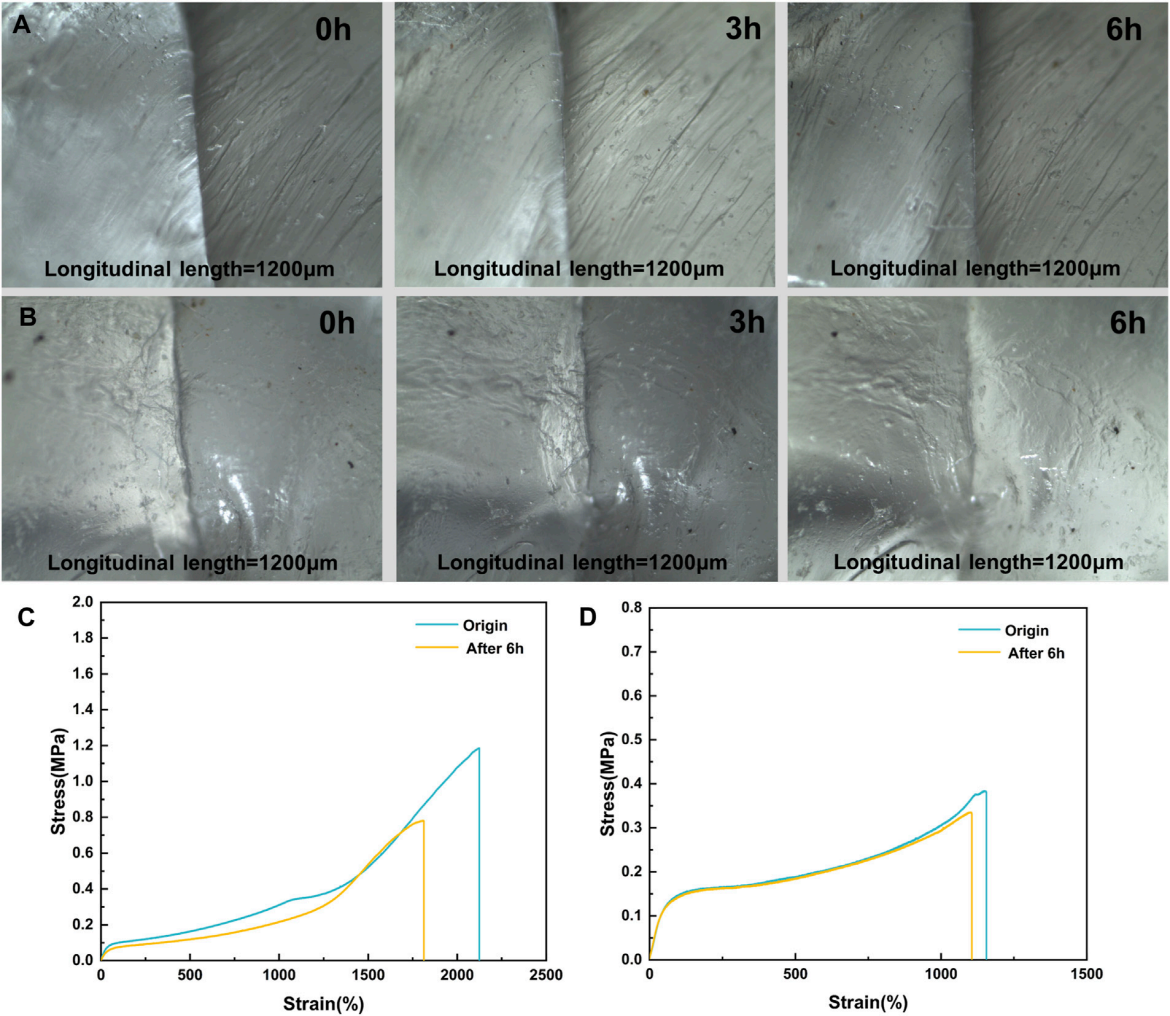
**(A,B)** Optical Images of Scratch Self-healing and **(C,D)** Tensile Properties Before and After Repair for M_1_ and M_3_.

The physical cross-linking network inherent in elastomers not only imparted them with shape memory functionality but also endowed them with self-healing properties. The self-heal capacity of elastomers relies on the reversible dynamics of hydrogen bonding and the predominance of π-π stacking interactions. We have examined the self-heal capability of elastomers through meticulous analysis using optical microscopy and precise tensile testing. The self-healing phenomena depicted in Figures 7A, B vividly showcased the remarkable self-healing attributes of the elastomer. Upon bisecting the elastomer into two segments, optimal self-healing was achieved by carefully aligning and adhering the wounded surfaces without external pressure, followed by a 37°C incubation for 6 h. Leveraging its inherent shape memory function, the elastomer could substantially restore the original configuration of the damaged site through stored strain, maximizing the contact area during the repair process. Upon contact, the fractured hydrogen bonding network underwent a reconstructive phase, enabling the healing of the elastomer wound.

In addition to examining the macroscopic morphology, it was crucial to consider the recovery of elastomer mechanical properties. By analyzing stress-strain curves of the original samples following self-healing, Figures 7C, D illustrated that after 6 h of self-healing at 37°C, the fracture stress of M_3_ was restored to 87.6% of its initial value, whereas M_1_ only recovered to 65.8% of its initial value. The study demonstrated that the self-healing ability of M_3_ surpasses that of M_1_. This was attributable to the higher MBC content found in M_3_ than in M_1_. As a result, there was a greater likelihood and number of hydrogen bonding and π-π stacking formation, which enhanced the self-healing process. The excellent room temperature self-repairing properties and shape memory at body temperature of the elastomers gave them the potential to be prepared as self-retracting surgical suture money, which was further investigated in the clinic.

## 4 Conclusion

Through the copolymerization of MBC and ε-CL with mPEG_43_ as the initiator and Sn(Oct)_2_ as the catalyst, novel smart elastomers, denoted as mPEG-*b*-(PMBC-co-PCL)_n_, were successfully synthesized. These elastomers exhibited exceptional mechanical properties, demonstrated by a tensile ratio of 1,155.6% and a tensile strength of 0.38 MPa. Notably, a remarkable shape recovery of 93% was achieved within 32.5 s at 37°C, underscoring their efficient shape memory performance. Moreover, their self-healing capability, primarily driven by hydrogen bonding and π-π stacking interactions, showcased an impressive self-healing efficiency of 87.6% after a 6 h self-healing process at room temperature without external stimuli. The elastomer mPEG-*b*-(PMBC-co-PCL)_n_ boasted exceptional attributes, including its near insolubility in water, remarkable shape memory capabilities, and outstanding self-heal efficiency. These unique qualities opened up possibilities for its utilization in the realm of self-retracting surgical sutures, potentially revolutionizing the field of clinical surgical procedures.

## Data Availability

The raw data supporting the conclusion of this article will be made available by the authors, without undue reservation.

## References

[B1] Alshomer F. Madhavan A. Pathan O. Song W. (2017). Bioactive sutures: a review of advances in surgical suture functionalisation. Curr. Med. Chem. 24 (2), 215–223. 10.2174/0929867324666161118141724 27855619

[B2] Ansari I. Singh P. Mittal A. Mahato R. I. Chitkara D. (2021). 2,2-Bis(hydroxymethyl) propionic acid based cyclic carbonate monomers and their (co)polymers as advanced materials for biomedical applications. Biomaterials 275, 120953. 10.1016/j.biomaterials.2021.120953 34218051

[B3] Bai J. Shi Z. (2017). Dynamically cross-linked elastomer hybrids with light-induced rapid and efficient self-healing ability and reprogrammable shape memory behavior. ACS Appl. Mater. Interfaces 9 (32), 27213–27222. 10.1021/acsami.7b06407 28745044

[B4] Chen C. Chen S. Guo Z. Hu W. Chen Z. Wang J. (2020). Highly efficient self-healing materials with excellent shape memory and unprecedented mechanical properties. J. Mater. Chem. A 8 (32), 16203–16211. 10.1039/d0ta04933f

[B5] Chen C. Hou Z. Chen S. Guo J. Chen Z. Hu J. (2022). Photothermally responsive smart elastomer composites based on aliphatic polycarbonate backbone for biomedical applications. Compos. Part B Eng. 240, 109985. 10.1016/j.compositesb.2022.109985

[B6] Chen C. Li Z. Chen S. Kong L. Guo Z. Hu J. (2021b). The preparation of hydrogels with highly efficient self-healing and excellent mechanical properties. J. Mol. Liq. 329, 115581. 10.1016/j.molliq.2021.115581

[B7] Chen Z. Li Y. Yao C. (2021a). Biomass shape memory elastomers with rapid self-healing properties and high recyclability. Biomacromolecules 22 (6), 2768–2776. 10.1021/acs.biomac.1c00465 34033462

[B8] Cui Y. Li D. Gong C. Chang C. (2021). Bioinspired shape memory hydrogel artificial muscles driven by solvents. ACS nano 15 (8), 13712–13720. 10.1021/acsnano.1c05019 34396782

[B9] Danhier F. Magotteaux N. Ucakar B. Lecouturier N. Brewster M. Préat V. (2009). Novel self-assembling PEG-p-(CL-co-TMC) polymeric micelles as safe and effective delivery system for paclitaxel. Eur. J. Pharm. Biopharm. 73 (2), 230–238. 10.1016/j.ejpb.2009.06.015 19577643

[B10] Davidson E. C. Kotikian A. Li S. Aizenberg J. Lewis J. A. (2020). 3D printable and reconfigurable liquid crystal elastomers with light‐induced shape memory via dynamic bond exchange. Adv. Mater. 32 (1), 1905682. 10.1002/adma.201905682 31664754

[B11] Duarah R. Singh Y. P. Gupta P. Mandal B. B. Karak N. (2018). Smart self-tightening surgical suture from a tough bio-based hyperbranched polyurethane/reduced carbon dot nanocomposite. Biomed. Mater. 13 (4), 045004. 10.1088/1748-605x/aab93c 29570096

[B12] Fan C.-J. Huang Z.-C. Li B. Xiao W.-X. Zheng E. Yang K.-K. (2019). A robust self-healing polyurethane elastomer: from H-bonds and stacking interactions to well-defined microphase morphology. Sci. China Mater. 62 (8), 1188–1198. 10.1007/s40843-019-9422-7

[B13] Gallos A. Crowet J.-M. Michely L. Raghuwanshi V. S. Mention M. M. Langlois V. (2021). Blending ferulic acid derivatives and polylactic acid into biobased and transparent elastomeric materials with shape memory properties. Biomacromolecules 22 (4), 1568–1578. 10.1021/acs.biomac.1c00002 33689317

[B14] Guo H. Han Y. Zhao W. Yang J. Zhang L. (2020). Universally autonomous self-healing elastomer with high stretchability. Nat. Commun. 11 (1), 2037. 10.1038/s41467-020-15949-8 32341363 PMC7184568

[B15] Han X. J. Dong Z. Q. Fan M. M. Liu Y. li J. H. Wang Y. F. (2012). pH‐induced shape‐memory polymers. Macromol. rapid Commun. 33 (12), 1055–1060. 10.1002/marc.201200153 22517685

[B16] Jian X. Hu Y. Zhou W. Xiao L. (2018). Self‐healing polyurethane based on disulfide bond and hydrogen bond. Polym. Adv. Technol. 29 (1), 463–469. 10.1002/pat.4135

[B17] Jiang Z.-C. Xiao Y.-Y. Kang Y. Pan M. Li B.-J. Zhang S. (2017). Shape memory polymers based on supramolecular interactions. ACS Appl. Mater. interfaces 9 (24), 20276–20293. 10.1021/acsami.7b03624 28553712

[B18] Kanu N. J. Gupta E. Vates U. K. Singh G. K. (2019). Self-healing composites: a state-of-the-art review. Compos. Part A Appl. Sci. Manuf. 121, 474–486. 10.1016/j.compositesa.2019.04.012

[B19] Kong D. Li J. Guo A. Zhang X. Xiao X. (2019). Self-healing high temperature shape memory polymer. Eur. Polym. J. 120, 109279. 10.1016/j.eurpolymj.2019.109279

[B20] Lapcık L. Jr Lapcık L. De Smedt S. Demeester J. Chabrecek P. (1998). Hyaluronan: preparation, structure, properties, and applications. Chem. Rev. 98 (8), 2663–2684. 10.1021/cr941199z 11848975

[B21] Li Z. Shan Y. Wang X. Li H. Yang K. Cui Y. (2020). Self-healing flexible sensor based on metal-ligand coordination. Chem. Eng. J. 394, 124932. 10.1016/j.cej.2020.124932

[B22] Liu Y. Lv H. Lan X. Leng J. Du S. (2009). Review of electro-active shape-memory polymer composite. Compos. Sci. Technol. 69 (13), 2064–2068. 10.1016/j.compscitech.2008.08.016

[B23] Liu Z. L. Zhou Y. Zhuo R. X. (2003). Synthesis and properties of functional aliphatic polycarbonates. J. Polym. Sci. Part A Polym. Chem. 41 (24), 4001–4006. 10.1002/pola.11001

[B24] Mathot F. des Rieux A. Arien A. Schneider Y.-J. Brewster M. Préat V. (2007). Transport mechanisms of mmePEG750P (CL-co-TMC) polymeric micelles across the intestinal barrier. J. Control. release 124 (3), 134–143. 10.1016/j.jconrel.2007.09.001 17928087

[B25] Pires L. R. Guarino V. Oliveira M. J. Ribeiro C. C. Barbosa M. A. Ambrosio L. (2016). Ibuprofen‐loaded poly (trimethylene carbonate‐co‐ε‐caprolactone) electrospun fibres for nerve regeneration. J. tissue Eng. Regen. Med. 10 (3), E154–E166. 10.1002/term.1792 23950030

[B26] Qi X. Yao X. Deng S. Zhou T. Fu Q. (2014). Water-induced shape memory effect of graphene oxide reinforced polyvinyl alcohol nanocomposites. J. Mater. Chem. A 2 (7), 2240–2249. 10.1039/c3ta14340f

[B27] Shao K. Han B. Gao J. Jiang Z. Liu W. Liu W. (2016). Fabrication and feasibility study of an absorbable diacetyl chitin surgical suture for wound healing. J. Biomed. Mater. Res. Part B Appl. Biomaterials 104 (1), 116–125. 10.1002/jbm.b.33307 25677094

[B28] Sheng Z.-Z. Liu X. Min L.-L. Wang H.-L. Liu W. Wang M. (2017). Bioinspired approaches for medical devices. Chin. Chem. Lett. 28 (6), 1131–1134. 10.1016/j.cclet.2017.03.033

[B29] Song Y. Liu Y. Qi T. Li G. L. (2018). Towards dynamic but supertough healable polymers through biomimetic hierarchical hydrogen‐bonding interactions. Angew. Chem. Int. Ed. 57 (42), 13838–13842. 10.1002/anie.201807622 30144244

[B30] Tsukamoto Y. Oshima H. Katsumori T. Hamaguchi H. Yamamoto S. Iwanaga T. (2018). Endoscopic topical therapy using mesh for refractory suture failure after rectal cancer surgery. Cancer & Chemother. 45 (3), 474–476. 29650909

[B31] Utrera-Barrios S. Verdejo R. López-Manchado M. Á. Santana M. H. (2022). The final frontier of sustainable materials: current developments in self-healing elastomers. Int. J. Mol. Sci. 23 (9), 4757. 10.3390/ijms23094757 35563147 PMC9101787

[B32] Wang S. Urban M. W. (2020). Self-healing polymers. Nat. Rev. Mater. 5 (8), 562–583. 10.1038/s41578-020-0202-4

[B33] Wang X. Liang D. Cheng B. (2020). Preparation and research of intrinsic self-healing elastomers based on hydrogen and ionic bond. Compos. Sci. Technol. 193, 108127. 10.1016/j.compscitech.2020.108127

[B34] Wemyss A. M. Bowen C. Plesse C. Vancaeyzeele C. Nguyen G. T. Vidal F. (2020). Dynamic crosslinked rubbers for a green future: a material perspective. Mater. Sci. Eng. R Rep. 141, 100561. 10.1016/j.mser.2020.100561

[B35] White S. R. Sottos N. R. Geubelle P. H. Moore J. S. Kessler M. R. Sriram S. (2001). Autonomic healing of polymer composites. Nature 409 (6822), 794–797. 10.1038/35057232 11236987

[B36] Wu W. Zhou Y. Li J. Wan C. (2020). Shape memory and self‐healing behavior of styrene–butadiene–styrene/ethylene‐methacrylic acid copolymer (SBS/EMAA) elastomers containing ionic interactions. J. Appl. Polym. Sci. 137 (19), 48666. 10.1002/app.48666

[B37] Xie Z. Hu B.-L. Li R.-W. Zhang Q. (2021). Hydrogen bonding in self-healing elastomers. ACS omega 6 (14), 9319–9333. 10.1021/acsomega.1c00462 33869912 PMC8047772

[B38] Xu J. Feng E. Song J. (2014). Renaissance of aliphatic polycarbonates: new techniques and biomedical applications. J. Appl. Polym. Sci. 131 (5). 10.1002/app.39822 PMC4076343 24994939

[B39] Yang L. Li J. Jin Y. Zhang J. Li M. Gu Z. (2014b). Highly efficient cross-linking of poly(trimethylene carbonate) via bis(trimethylene carbonate) or bis(ε-caprolactone). Polymer 55 (26), 6686–6695. 10.1016/j.polymer.2014.10.072

[B40] Yang L. Li J. Meng S. Jin Y. Zhang J. Li M. (2014a). The *in vitro* and *in vivo* degradation behavior of poly (trimethylene carbonate-co-ε-caprolactone) implants. Polymer 55 (20), 5111–5124. 10.1016/j.polymer.2014.08.027

[B41] Yang S. Du X. Deng S. Qiu J. Du Z. Cheng X. (2020). Recyclable and self-healing polyurethane composites based on Diels-Alder reaction for efficient solar-to-thermal energy storage. Chem. Eng. J. 398, 125654. 10.1016/j.cej.2020.125654

[B42] Yin C. Wang T. Shen X. Fu J. Li T. Jiang T. (2022). Body-temperature programmable ultra-soft shape memory elastomers for comfort fitting. Smart Mater. Struct. 31 (10), 105029. 10.1088/1361-665x/ac9101

[B43] Ze Q. Kuang X. Wu S. Wong J. Montgomery S. M. Zhang R. (2020). Magnetic shape memory polymers with integrated multifunctional shape manipulation. Adv. Mater. 32 (4), 1906657. 10.1002/adma.201906657 31814185

[B44] Zhao W. Liu Y. Zhang Z. Feng X. Xu H. Xu J. (2020). High-strength, fast self-healing, aging-insensitive elastomers with shape memory effect. ACS Appl. Mater. interfaces 12 (31), 35445–35452. 10.1021/acsami.0c09045 32643374

[B45] Zhao X. Dong R. Guo B. Ma P. X. (2017). Dopamine-incorporated dual bioactive electroactive shape memory polyurethane elastomers with physiological shape recovery temperature, high stretchability, and enhanced C2C12 myogenic differentiation. ACS Appl. Mater. interfaces 9 (35), 29595–29611. 10.1021/acsami.7b10583 28812353

